# Somatic health care professionals’ stigmatization of patients with mental disorder: a scoping review

**DOI:** 10.1186/s12888-021-03415-8

**Published:** 2021-09-07

**Authors:** Ida Nielsen Sølvhøj, Amalie Oxholm Kusier, Pia Vivian Pedersen, Maj Britt Dahl Nielsen

**Affiliations:** National Institute of Public Health, University of Southern Denmark, Studiestræde 6, DK-1455 Copenhagen, Denmark

**Keywords:** Mental health, Mental disorders, Scoping review, Health care professional, Health care student, Stigma

## Abstract

**Background:**

Patients with mental disorders have an increased risk of developing somatic disorders, just as they have a higher risk of dying from them. These patients often report feeling devaluated and rejected by health professionals in the somatic health care system, and increasing evidence shows that disparities in health care provision contribute to poor health outcomes. The aim of this review was to map and synthesize literature on somatic health professionals’ stigmatization toward patients with mental disorders.

**Methods:**

We conducted a scoping review using Arksey and O’Malley’s framework and carried out a systematic search in three databases: Cinahl, MEDLINE, and PsycINFO in May–June 2019. Peer-reviewed articles published in English or Scandinavian languages during 2008–2019 were reviewed according to title, abstract and full-text reading. We organized and analyzed data using NVivo.

**Results:**

A total of 137 articles meeting the eligibility criteria were reviewed and categorized as observational studies (*n* = 73) and intervention studies (*n* = 64). A majority of studies (*N* = 85) focused on patients with an unspecified number of mental disorders, while 52 studies focused on specific diagnoses, primarily schizophrenia (*n* = 13), self-harm (n = 13), and eating disorders (*n* = 9). Half of the studies focused on health students (*n* = 64), primarily nursing students (*n* = 26) and medical students (*n* = 25), while (*n* = 66) focused on health care professionals, primarily emergency staff (*n* = 16) and general practitioners (*n* = 13). Additionally, seven studies focused on both health professionals and students. A detailed characterization of the identified intervention studies was conducted, resulting in eight main types of interventions.

**Conclusions:**

The large number of studies identified in this review suggests that stigmatizing attitudes and behaviors toward patients with mental disorders is a worldwide challenge within a somatic health care setting. For more targeted interventions, there is a need for further research on underexposed mental diagnoses and knowledge on whether specific health professionals have a more stigmatizing attitude or behavior toward specific mental disorders.

**Supplementary Information:**

The online version contains supplementary material available at 10.1186/s12888-021-03415-8.

## Background

Mental disorders, such as anxiety, depression and substance use disorders, are among the most burdensome disorders in the world [1, 2], and the estimated life expectancy is generally 15–20 years lower for patients with mental disorders compared to the general population [3]. Research shows that somatic disorders are the main cause of this excess mortality [4] and respiratory, digestive and cardiovascular diseases account for the largest impact on mortality among patients with schizophrenia [5]. Not only do patients with mental disorders have a higher risk of developing somatic disorders, they also have a higher risk of dying from them [3, 6–8]. While numerous factors affect the morbidity and mortality of this patient group, increasing evidence shows that disparities in health care provision contribute to poor health outcomes [9–12]. ‘Diagnostic overshadowing’ – the misattribution of physical symptoms to mental illness – is a key concept used in many studies to describe these disparities, as it can contribute to treatment delay and the development of complications [13–15].

In this review, we argue that stigma is key to understanding these disparities. First, stigma can affect multiple life domains and probably has a dramatic bearing on the distribution of life chances in a variety of areas such as earnings, housing and criminal involvement [12]. Second, patients with mental disorders often report feeling devaluated and rejected by health professionals [13, 16], and third, previous research shows that stigma affects patients’ willingness to seek treatment and the quality of care [17–20].

Stigma is a complex phenomenon, and definitions vary across disciplines and research fields. According to Link et al. (2001) researchers criticize the term for being too vaguely defined and individually focused. In response to this criticism, Link et al. (2001) proposed a new definition highlighting that: “Stigma exists when elements of labeling, stereotyping, separation, status loss, and discrimination occur together in a power situation that allows them*”* [12]. This paper focuses specifically on stigma in the somatic health care system toward patients with mental disorders, because suboptimal treatment of serious and potentially life-threatening somatic conditions can have profound negative implications for the patients. Previous literature on mental disorder stigma within somatic health care typically focuses on specific mental disorders or specific health professions and settings [8, 21–24]. Thus, a general overview is lacking. This review aims to provide an overview of the literature on stigmatization among somatic health care professionals toward patients with mental disorders—across different health care professions and mental disorders. More specifically, we aim to: 1) provide an overall characterization of existing observational and intervention studies according to health care profession and diagnosis, 2) provide a detailed characterization of the identified intervention studies, and 3) identify knowledge gaps.

## Method

Scoping reviews aim to create an overview of a research field and to give an indication of the volume of the literature. Furthermore, a scoping review can be helpful in identifying knowledge gaps [25]. The process of conducting a scoping review is systematic and structured [26] and to ensure transparency, we were informed by the PRISMA-ScR guidelines [27] and Arksey and O’Malley’s methodological framework [28], which outlines six stages of conducting scoping reviews.

### Stage 1: identification of research question

In health research, it has become increasingly common to engage with stakeholders such as policy makers; clinicians; and patients, just as many research institutions offers research-based collaboration and advice to external parties. These collaborations can help improve study questions and provide more useful findings [29]. In the design of the literature study, we collaborated with The Danish Health Authority and the organization EN AF OS, who aims to destigmatize mental illness in Denmark. They were both involved in developing the research question, and the organization was additionally involved in qualifying keywords and search strings. As recommended by Arksey and O’Malley, we both considered relevant aspects of the research question (e.g. study population, phenomenon of interest and context) and were aware of developing a research question with a wide approach in order to generate breadth of coverage [28].

### Stage 2: identifying relevant data

The first author developed the search strategy in collaboration with research librarians. The search strategy was based on the PICo model that specifies the population (P), phenomenon of interest (I), and context (Co). Following the PICo model the search consisted of two search strings. The first search string specified the population (P), which included patients with frequently occurring mental disorders. We used broad search terms such as ‘mental disorders’ and additionally performed searches on seven specific disorders, since a too broad search can result in missing relevant studies. The seven specific disorders included: anxiety, depression, bipolar disorder, borderline, schizophrenia, eating disorders, and self-harm. We chose these, because they are among the most common and disabling mental disorders [2]. The second search string specified the phenomenon of interest (I) and context (Co), which included stigmatizing behaviors, attitudes, and perceptions among health professionals, e.g. nurses and physicians, in the somatic health care system (see Additional file 1 for full search string). The search was based on keywords (subject headings/ MeSH terms) and free text searches (title, keywords, text). We tested the search string before we formed the final search string. To delimit the search, we applied proximity searching of two words. The search was carried out in May 2019 in three databases: Cinahl, MEDLINE and PsycINFO. Title, abstract and full paper screening were based on inclusion and exclusion criteria. To narrow down the search, we only included studies published 2008–2019 in peer-reviewed journals in English or Scandinavian languages. The temporal delineation was made as we wanted to find the most recent published literature in the field. Protocols, conference literature, book chapters, opinion papers and reviews were excluded. We excluded studies focusing on the mental health of health professionals as well as studies where somatic and psychiatric health professionals could not be separated. While we only included observational studies from the Western Hemisphere, we applied no geographical exclusion criteria for the intervention studies. This is because we wanted to gain insight into experiences from interventions conducted all over the world and because the wide range of interventions may contribute as inspiration for future prevention work. We made the choice of only including observational studies from the Western Hemisphere in collaboration with the organization EN AF OS, to narrow down the search.

### Stage 3: data selection

**Fig. 1:**
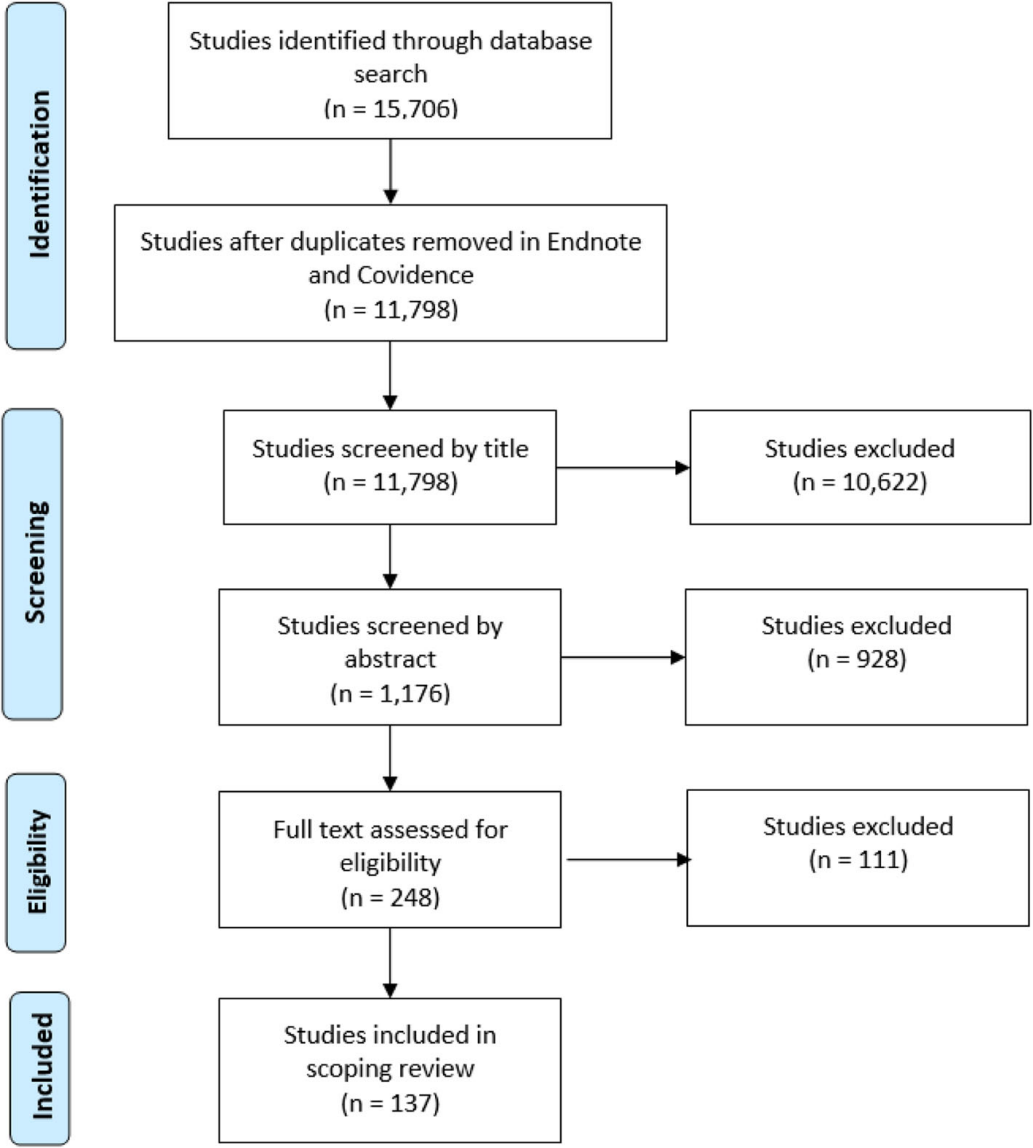
Flow Diagram

We stored the studies in Endnote and removed duplicates. Next, we moved the studies to the review manager Covidence and performed another duplicate check, after which we began the screening process of the 11,798 identified studies. During the initial title screening, we excluded 10,622 studies for being out of scope. Additionally, we excluded 928 studies during abstract screening for not meeting the inclusion criteria. Of the remaining 248 studies, 111 studies were excluded for not meeting the inclusion criteria. Finally, we included a total of 137 studies in the scoping review. Figure 1 summarizes the literature search and study selection.

### Stage 4 and 5: charting and collating data

We used NVivo to organize and analyze the data. The data charting took the following information into consideration: author(s), year of publication, country of origin, study design, study population (health professionals in the somatic health system), sample size, and mental disorders. Additionally, we coded the scope, method, intervention type, instrument, and outcome in each study. Subsequently, we summarized the studies by study design: observational studies and intervention studies.

### Stage 6: consultation exercise

As briefly described above, we collaborated with The Danish Health Authority and the organization EN AF OS, in the development of the design of the literature review and of the research question. The organization was additionally involved in the process of further qualifying keywords and search strings to ensure that relevant keywords, including mental disorders and specific health professionals were not omitted. Thus, the organization played an important role in quality assuring the first two stages of the scoping review. Further, we discussed the findings of the scoping review with the organization and learned that our findings corresponded well with the organization’s knowledge of the phenomenon.

## Results

In total, we included 137 studies of which 73 were observational studies and 64 were intervention studies. In the following, we describe and categorize the identified studies according to diagnosis and health care profession. All included studies examined stigmatizing behaviors, attitudes, and/or perceptions among health professionals in the somatic health care system toward patients with mental disorders. For the sake of readability, we will primarily refer to this information as ‘attitudes and behaviors’.

### Categorization of health professionals and patients

**Fig. 2:**
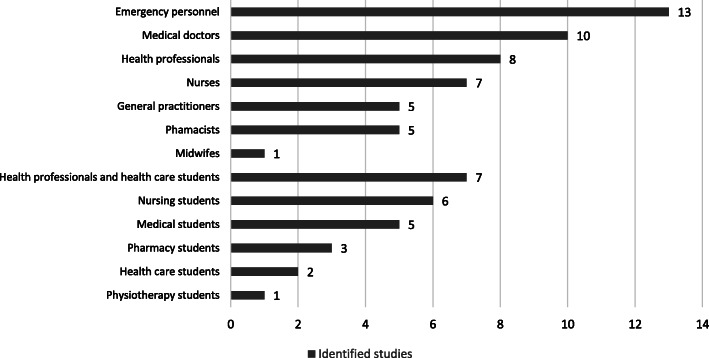
Identified observational studies categorized according to health profession

To create an overview of the wide range of different health professions included in the identified studies, we divided the health professionals into 13 categories (Fig. 2). Some studies focused on students rather than trained professionals, and we categorized these studies separately. Other studies included both students and professionals. Moreover, while some studies included well-defined groups of health professionals, such as nurses or general practitioners, others did not focus on specific health professionals. These studies were categorized under the heading ‘Health professionals’, e.g. various hospital employees. Similarly, the category ‘Health care students’ refers to various students within health education. However, we also identified studies examining both students and health professionals, which formed the broad category ‘Health professionals and health care students’. Finally, the category ‘Medical doctors’ covers all other types of medical doctors besides general practitioners, e.g. surgeons or different types of medical specialists.

We also categorized the identified studies according to the patients’ diagnoses and formed seven categories. Five of the categories cover specific diagnoses. In addition, we included a ‘Mixed mental disorders’ category. This category includes studies not confined to a specific mental disorder or studies that examined multiple diagnoses. Furthermore, the category ‘Dual diagnosis’ covers studies examining patients with a mental disorder and a substance use disorder.

### Characteristics of the observational studies

The main purpose of the observational studies was to investigate the magnitude of stigmatizing attitudes and behaviors among health professionals in the somatic health care system toward patients with mental disorders. In total, we identified 73 observational studies, all from the Western Hemisphere. About half of the studies were from Europe (*n* = 41), including a large proportion of studies from England (*n* = 16), followed by North America (*n* = 15), Oceania (*n* = 11), and Asia (*n* = 3). Furthermore, we identified three studies comparing populations across countries. Most studies used quantitative methods (*n* = 58); however, we also identified qualitative studies (*n* = 13) and mixed methods studies (*n* = 2).

As illustrated in Fig. 2, we found that most studies (n = 13) focused on emergency personnel, followed by medical doctors (*n* = 10) and health professionals (*n* = 8). Most studies (*n* = 40) focused on several, different diagnoses (Table 1). In studies focusing on single, specific diagnoses, the most frequent mental disorders were self-harm (n = 10) and schizophrenia (*n* = 9).

We combined type of health profession with diagnosis as shown in Table 1. The table shows that a relatively large proportion of studies examined attitudes and behaviors among emergency staff toward patients who self-harm (*n* = 6). In contrast, studies examining attitudes and behaviors among medical doctors (*n* = 10) were divided into a wide range of diagnoses, such as depression (n = 1), schizophrenia (n = 1), eating disorders (*n* = 2), self-harm (n = 2), mixed mental disorders (*n* = 3), and dual diagnosis (n = 1).

**Table 1:** Combination of diagnoses and health care profession (observational studies)

Diagnosis	Health care profession	Number of studies
Depression (n = 3)	Health care students	1
	Pharmacists	1
	Medical doctors	1
Borderline (n = 2)	Nurses	1
	Emergency personnel	1
Schizophrenia (n = 9)	Health professionals and health care students	2
	Nursing students	1
	Pharmacy students	2
	Pharmacists	1
	Medical students	1
	Medical doctors	1
	General practitioners	1
Eating disorder (n = 5)	Health care students	1
	Health professionals	1
	Medical doctors	2
	General practitioners	1
Self-harm (n = 10)	Health professionals	2
	Medical doctors	2
	Emergency personnel	6
Mixed mental disorders (n = 40)	Physiotherapy students	1
	Midwifes	1
	Health professionals and health care students	5
	Nursing students	6
	Nurses	5
	Health professionals	3
	Pharmacy students	1
	Pharmacists	4
	Medical students	4
	Medical doctors	3
	General practitioners	3
	Emergency personnel	5
Dual diagnosis (n = 4)	Health professionals	2
	Medical doctors	1
	Emergency personnel	1
**Total**		**73**

For a detailed description of study design, target group, sample size and diagnosis on observational studies, see Table 2.

**Table 2:** Observational studies

First author, year, reference	Country	Population	Sample size	Diagnosis	Design
Abood, 2009 [30]	UK	Medical doctors	*N* = 47	Self-harm	Quantitative
Anderson, 2017 [31]	USA	Medical doctors	*N* = 80	Eating disorder	Quantitative
Arbanas, 2019 [32]	Croatia	Health professionals	*N* = 387	Mixed	Quantitative
Artis, 2013 [33]	UK	Emergency personnel	N = 10	Self-harm	Qualitative
Arvaniti, 2009 [34]	Greece	Health professionals and health care students	*N* = 592	Mixed	Quantitative
Avery, 2019 [35]	USA	Medical doctors	*N* = 411	Dual diagnosis	Quantitative
Bannatyne, 2017 [36]	Australia	Health care students	*N* = 126	Eating disorder	Quantitative
Bell, 2010 [37]	Australia, Belgium, India, Finland, Estonia, Latvia	Pharmacy students	*N* = 649	Schizophrenia	Quantitative
Bell, 2008 [38]	Australia, Belgium, India, Finland, Estonia, Latvia	Pharmacy students	*N* = 642	Mixed	Quantitative
Bjorkman, 2008 [39]	Sweden	Nurses	*N* = 120	Mixed	Quantitative
Brunero, 2017 [40]	Australia	Nurses	N = 16	Mixed	Qualitative
Castillejos, 2019 [41]	Spain	General practitioners	*N* = 145	Mixed	Quantitative
Ceylan, 2019 [42]	Turkey	Nurses	*N* = 186	Schizophrenia	Quantitative
Chapman, 2014 [43]	Australia	Emergency Personnel	N = 186	Self-harm	Quantitative
Clifton, 2016 [44]	UK	Health professionals	N = 85	Mixed	Qualitative
Conlon, 2012 [45]	Ireland	Emergency personnel	*N* = 87	Self-harm	Quantitative
Crapanzano, 2018 [46]	USA	Medical doctors	*N* = 96	Depression	Quantitative
Currin, 2009 [47]	UK	General practitioners	*N* = 154	Eating disorders	Quantitative
Cutler, 2009 [48]	USA	Medical students	N = 47	Mixed	Qualitative
Dixon, 2008 [49]	UK	Medical students	*N* = 1081	Mixed	Quantitative
Ewalds-Kvist, 2013 [50]	Sweden	Nursing students	*N* = 246	Mixed	Quantitative
Gawley, 2011 [51]	Canada	Health care students	*N* = 309	Depression	Quantitative
Giannetti, 2018 [52]	USA	Pharmacists	*N* = 239	Mixed	Quantitative
Gordon, 2012 [53]	UK	Emergency personnel	*N* = 32	Mixed	Quantitative
Granados-Gamez, 2017 [54]	Spain	Nursing students	*N* = 194	Mixed	Quantitative
Happell, 2008 [55]	Australia	Nursing students	*N* = 148	Mixed	Quantitative
Happell, 2018 [56]	Australia, Ireland, Finland, Norway, Netherland	Nursing students	*N* = 423	Mixed	Quantitative
Heyward-Chaplin, 2018 [57]	UK	Health professionals	N = 59	Self-harm	Quantitative
Ihalainen-Tamlander, 2016 [58]	Finland	Nurses	*N* = 218	Mixed	Quantitative
Janouskova, 2017 [59]	The Czech Republic	Health professionals and health care students	*N* = 308	Mixed	Quantitative
Jones, 2009 [60]	USA	Medical doctors	*N* = 51	Mixed	Quantitative
Koning, 2018 [61]	Australia	Emergency personnel	N = 15	Self-harm	Qualitative
Thongpriwan, 2015 [62]	USA	Nursing students	*N* = 229	Mixed	Quantitative
Kopera, 2015 [63]	Polonia	Health professionals and health care students	*N* = 57	Mixed	Quantitative
Korszun, 2012 [64]	UK	Medical students	*N* = 760	Mixed	Quantitative
Kuzman, 2014 [65]	The Czech Republic	Medical students	*N* = 199	Mixed	Quantitative
Leddy, 2009 [66]	USA	Medical doctors	*N* = 504	Eating disorder	Quantitative
Liekens, 2012 [67]	Belgium	Pharmacists	*N* = 149	Depression	Quantitative
Magliano, 2011 [68]	Italy	Medical students	N = 194	Schizophrenia	Quantitative
Magliano, 2017 [69]	Italy	General practitioners	*N* = 387	Schizophrenia	Quantitative
McCann, 2018 [70]	Australia	Emergency personnel	*N* = 1230	Dual diagnosis	Quantitative
McCarthy, 2010 [71]	Ireland	Emergency Personnel	*N* = 68	Self-harm	Quantitative
Morral, 2016 [72]	UK	Pharmacists	*N* = 351	Mixed	Quantitative
Muehlenkamp, 2013 [73]	Belgium	Health professionals	*N* = 342	Self-harm	Quantitative
Nash, 2013 [13]	UK	Emergency personnel	*N* = 39	Mixed	Qualitative
Nauta, 2019 [74]	Netherlands	Medical doctors	*N* = 187	Mixed	Quantitative
Neauport, 2012 [75]	France	Medical doctors	*N* = 322	Mixed	Quantitative
Noonan, 2018 [76]	Ireland	Midwifes	*N* = 157	Mixed	Quantitative
Nutt, 2017 [77]	Scotland	Health professionals	*N* = 113	Dual diagnosis	Quantitative
O’Reilly, 2012 [78]	Australia	Health professionals and health care students	N = 23	Mixed	Qualitative
O’Reilly, 2015 [79]	Australia	Pharmacists	*N* = 188	Schizophrenia	Quantitative
Peitl, 2011 [80]	Croatia	Health professionals and health care students	*N* = 151	Mixed	Quantitative
Perboell, 2015 [81]	Denmark	Emergency personnel	*N* = 122	Self-harm	Quantitative
Prener, 2015 [82]	USA	Emergency personnel	N = 20	Mixed	Qualitative
Rai, 2019 [83]	UK	Medical doctors	*N* = 37	Self-harm	Mixed methods
Rao, 2009 [84]	UK	Health professionals	N = 108	Dual diagnosis	Quantitative
Raveneau, 2014 [85]	USA	Health professionals	*N* = 82	Eating disorder	Quantitative
Reavley, 2014 [86]	Australia	Health professionals	*N* = 1536	Mixed	Quantitative
Rickles, 2010 [87]	USA	Pharmacists	*N* = 292	Mixed	Quantitative
Sandhu, 2019 [88]	Canada	Health professionals and health care students	*N* = 538	Schizophrenia	Quantitative
Schafer, 2011 [89]	UK	Nursing students	*N* = 288	Mixed	Quantitative
Schmidt, 2017 [90]	Netherlands	General practitioners	*N* = 63	Mixed	Quantitative
Serafini, 2011 [91]	Italy	Health professionals and health care students	*N* = 202	Schizophrenia	Quantitative
Shefer, 2014 [15]	UK	Emergency personnel	N = 39	Mixed	Qualitative
Stumbo, 2018 [92]	USA	General practitioners	*N* = 597	Mixed	Mixed methods
Treloar, 2009 [93]	Australia	Emergency personnel	*N* = 140	Borderline personality disorder	Qualitative
Van Nieuwenhui, 2013 [94]	UK	Emergency personnel	N = 25	Mixed	Qualitative
Volmer, 2008 [95]	Estonia	Pharmacy students	N = 157	Schizophrenia	Quantitative
Weare, 2019 [96]	Australia	Nurses	N = 40	Mixed	Quantitative
Winkler, 2016 [97]	The Czech Republic	Medical doctors	*N* = 3010	Mixed	Quantitative
Woollaston, 2008 [98]	UK	Nurses	N = 6	Borderline personality disorder	Qualitative
Yildirim, 2015 [99]	Turkey	Physiotherapy students	*N* = 524	Mixed	Quantitative
Zolnierek, 2012 [100]	USA	Nurses	N = 1	Mixed	Qualitative

### Characteristics of the intervention studies

The main purpose of the intervention studies was to evaluate interventions to reduce health professionals’ stigmatizing attitudes and behaviors toward people with mental disorders. We identified 64 intervention studies, most of which were from North America (*n* = 19), followed by studies from Europe (*n* = 16), Oceania (*n* = 13), Asia (n = 13), and Africa (n = 1). In addition, two interventions studies were comparative studies. Most studies were based on quantitative methods (*n* = 51); seven were mixed methods studies, and six were qualitative studies.

**Fig. 3:**
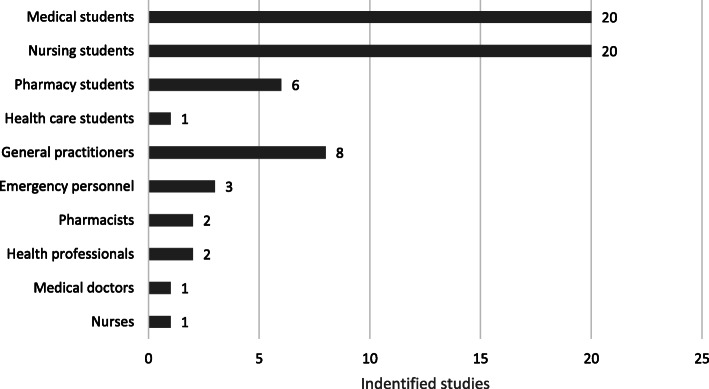
Identified intervention studies categorized according to health profession

Most intervention studies focused on changing students’ attitudes and behaviors toward patients with mental disorders; 20 of these studies focused on medical students, 20 focused on nursing students and six focused on pharmacy students, as illustrated in Fig. 3. Furthermore, eight studies focused on general practitioners, constituting the third-largest category when distributing the studies by health profession.

Most intervention studies (*n* = 45) did not focus on patients with a specific mental disorder, but typically on attitudes and behaviors toward multiple mental disorders or mental disorder in general as shown in Table 2. Depression (*n* = 5), eating disorder (n = 4), and schizophrenia (n = 4) were among the most common diagnoses.

By combining health profession with diagnosis (Table 3), we found that most of the studies focusing on attitudes and behaviors among nursing students did not focus on a specific mental disorder, as 17 of the 20 identified studies looked at multiple diagnoses or mental disorder in general. Similarly, 16 of 20 identified studies examining attitudes and behaviors among medical students focused on multiple diagnoses or mental disorder in general. We found a similar pattern for studies examining attitudes and behaviors among pharmacy students (*n* = 6) and health care students (*n* = 1).

**Table 3:** Combination of diagnoses and health care profession (intervention studies)

Diagnosis	Health care profession	Number of studies
Depression (*n* = 5)	General practitioners	3
	Pharmacists	2
Borderline (n = 2)	Emergency personnel	1
	Health professionals	1
Schizophrenia (n = 4)	Medical doctors	1
	Medical students	3
Eating disorder (n = 4)	General practitioners	1
	Medical students	1
	Nursing students	2
Self-harm (n = 3)	Emergency personnel	2
	Nursing students	1
Bipolar disorder (n = 1)	Health professionals	1
Mixed mental disorders (n = 45)	General practitioners	4
	Medical students	16
	Pharmacy students	6
	Health care students	1
	Nurses	1
	Nursing students	17
**Total**		**64**

For a detailed description of study design, target group, sample size, and diagnosis in the intervention studies, see Table 4.

**Table 4:** Intervention studies

First author, year	Country	Population	Sample size	Diagnosis	Design	Intervention
Airagnes, 2014 [101]	France	Medical students	*N* = 163	Mixed	Quasi-experimental with control group	Lectures
Arbanas, 2018 [102]	Croatia	Nursing students	N = 51	Mixed	Quasi-experimental without control group	Expeditionary interventions
Bannatyne, 2015 [103]	Australia	Medical students	N = 41	Eating disorder	Quasi-experimental with control group	Lectures
Beaulieu, 2017 [104]	Canada	General practitioners	*N* = 73	Mixed	RCT	Interventions targeting general practitioners and medical doctors
Bilge, 2017 [105]	Turkey	Nursing students	N = 322	Mixed	Other	Lectures
Bingham, 2018 [106]	New Zealand	Nursing students	N = 45	Mixed	Quasi-experimental without control group	Expeditionary interventions
Brenner, 2011 [107]	USA	Medical students	*N* = 100	Mixed	Qualitative	Expeditionary interventions
Calloway, 2017 [108]	USA	Nurses	N = 82	Mixed	Qualitative	Interventions targeting health professionals
Chiles, 2017 [109]	USA	Medical students	*N* = 289	Mixed	Quasi-experimental without control group	Observational studies
Clement, 2012 [110]	UK	Nursing students	*N* = 216	Mixed	RCT	Contact-based interventions
Coppens, 2018 [111]	Portugal, Germany, Ireland, Hungary	General practitioners	*N* = 208	Depression	Quasi-experimental without control group	Interventions targeting general practitioners and medical doctors
Crisafulli, 2008 [112]	USA	Nursing students	*N* = 115	Eating disorder	Quasi-experimental with control group	Lectures
Crockett, 2009 [113]	Australia	Pharmacists	N = 32	Depression	RCT	Interventions targeting pharmacists
Demiroren, 2016 [114]	Turkey	Medical students	*N* = 190	Mixed	Quasi-experimental with control group	Expeditionary interventions
Dipaula, 2011 [115]	USA	Pharmacy students	*N* = 278	Mixed	Quasi-experimental with control group	Lectures
Duffy, 2016 [116]	USA	Nursing students	*N* = 131	Eating disorder	Quasi-experimental without control group	Lectures
Duman, 2017 [117]	Turkey	Nursing students	N = 202	Mixed	Quasi-experimental with control group	Lectures
Economou, 2017 [118]	Greece	Medical students	*N* = 678	Mixed	Quasi-experimental without control group	Expeditionary interventions
Economou, 2012 [119]	Greece	Medical students	*N* = 158	Schizophrenia	Quasi-experimental without control group	Expeditionary interventions
Eksteen, 2017 [120]	The South African Republic	Medical students	*N* = 616	Mixed	Quasi-experimental without control group	Expeditionary interventions
Esen Danaci, 2016 [121]	Turkey	Medical students	*N* = 106	Schizophrenia	Quasi-experimental without control group	Observational studies
Failde, 2014 [122]	Spain	Medical students	*N* = 171	Mixed	Quasi-experimental without control group	Observational studies
Fernandez, 2016 [123]	Malaysia	Medical students	*N* = 102	Mixed	RCT	Contact-based interventions
Flanagan, 2016 [124]	USA	General practitioners	N = 27	Mixed	Quasi-experimental with control group	Interventions targeting general practitioners and medical doctors
Fokuo, 2017 [125]	USA	Nursing students	*N* = 70	Mixed	Qualitative	Contact-based interventions
Gable, 2011 [126]	USA	Pharmacy students	N = 39	Mixed	Quasi-experimental with control group	Lectures
Galletly, 2011 [127]	Australia	Medical students	N = 87	Schizophrenia	Quasi-experimental without control group	Contact-based interventions
Gibson, 2019 [128]	UK	Nursing students	*N* = 55	Self-harm	Quasi-experimental without control group	Lectures
Happell, 2008 [129]	Australia	Nursing students	*N* = 687	Mixed	Quasi-experimental without control group	Expeditionary interventions
Happell, 2019 [130]	Australia, Ireland, Finland	Nursing students	*N* = 194	Mixed	Quasi-experimental without control group	Contact-based interventions
Hastings, 2017 [131]	USA	Nursing students	*N* = 310	Mixed	Quasi-experimental without control group	Expeditionary interventions
Itzhaki, 2017 [132]	Israel	Nursing students	*N* = 101	Mixed	Quasi-experimental without control group	Contact-based interventions
Kassam, 2011 [133]	UK	Medical students	*N* = 110	Mixed	Quasi-experimental with control group	Contact-based interventions
Knaak, 2015 [134]	Canada	Health professionals	*N* = 191	Borderline personality disorder	Quasi-experimental without control group	Interventions targeting health professionals
Lam, 2011 [135]	Hong Kong	General practitioner	*N* = 69	Mixed	Quasi-experimental without control group	Interventions targeting general practitioners and medical doctors
Lam, 2015 [136]	Hong Kong	General practitioners	*N* = 566	Mixed	Quasi-experimental with control group	Interventions targeting general practitioners and medical doctors
Liekens, 2013 [137]	Belgium	Pharmacists	*N* = 141	Depression	RCT	Interventions targeting pharmacists
Linville, 2013 [138]	USA	General practitioners	N = 45	Eating disorder	Quasi-experimental without control group	Interventions targeting general practitioners and medical doctors
Lyons, 2015 [139]	Australia	Medical students	N = 151	Mixed	Quasi-experimental without control group	Expeditionary interventions
Manzanera, 2018 [140]	Spain	General practitioners	*N* = 1322	Depression	Quasi-experimental without control group	Interventions targeting general practitioners and medical doctors
Markstrom, 2009 [141]	Sweden	Health care students	*N* = 167	Mixed	Quasi-experimental without control group	Expeditionary interventions
Martinez-Martinez, 2019 [142]	Spain	Nursing students	*N* = 185	Mixed	Quasi-experimental without control group	Contact-based interventions
McAllister, 2009a [143]	Australia	Emergency personnel	N = 28	Self-harm	Quasi-experimental without control group	Interventions targeting emergency personnel
McAllister, 2009b [144]	Australia	Emergency personnel	*N* = 36	Self-harm	Quasi-experimental without control group	Interventions targeting emergency personnel
Michalak, 2014 [145]	Canada	Health professionals	*N* = 164	Bipolar disorder	Quasi-experimental without control group	Interventions targeting health professionals
Morrison, 2009 [146]	Australia	Nursing students	N/A	Mixed	Qualitative	Contact-based interventions
Moxham, 2016 [147]	Australia	Nursing students	N = 9	Mixed	Quasi-experimental with control group	Expeditionary interventions
Muzyk, 2017 [148]	USA	Pharmacy students	*N* = 74	Mixed	Quasi-experimental without control group	Lectures
O′ Connor, 2013 [149]	Ireland	Medical students	*N* = 285	Mixed	Quasi-experimental without control group	Expeditionary interventions
Omori, 2012 [150]	Japan	Medical doctors	N = 51	Schizophrenia	Quasi-experimental without control group	Interventions targeting general practitioners and medical doctors
O’Reilly, 2010 [151]	Australia	Pharmacy students	*N* = 178	Mixed	Quasi-experimental without control group	Contact-based interventions
O’Reilly, 2011 [152]	Australia	Pharmacy students	*N* = 60	Mixed	Quasi-experimental with control group	Lectures
Papish, 2013 [153]	Canada	Medical students	*N* = 111	Mixed	RCT	Expeditionary interventions
Patten, 2012 [154]	Canada	Pharmacy students	N = 131	Mixed	RCT	Contact-based interventions
Poreddi, 2015 [155]	India	Medical students	*N* = 176	Mixed	Quasi-experimental with control group	Lectures
Romem, 2008 [156]	Israel	Nursing students	N = 126	Mixed	Quasi-experimental without control group	Expeditionary interventions
Shen, 2014 [157]	China	Medical students	*N* = 325	Mixed	Quasi-experimental without control group	Expeditionary interventions
Stacey, 2018 [158]	UK	Nursing students	N/A	Mixed	Qualitative	Lectures
Stuhlmiller, 2019 [159]	USA	Nursing students	*N* = 85	Mixed	Quasi-experimental without control group	Expeditionary interventions
Telles-Correia, 2015 [160]	Portugal	Medical students	*N* = 398	Mixed	Quasi-experimental without control group	Observational studies
Treloar, 2009 [161]	Australia	Emergency personnel	*N* = 65	Borderline personality disorder	Quasi-experimental with control group	Interventions targeting emergency personnel
Upshur, 2008 [162]	USA	General practitioners	N = 9	Depression	Quasi-experimental without control group	Interventions targeting general practitioners and medical doctors
Wang, 2016 [163]	Taiwan	Medical students	*N* = 72	Mixed	Quasi-experimental with control group	Observational studies
Winkler, 2017 [164]	The Czech Republic	Nursing students	*N* = 499	Mixed	RCT	Contact-based interventions

Note: RCT = Randomized controlled trial, Observational studies = Observational studies of the effect of attending medical school

### Intervention types and content

Of the 64 included intervention studies, 47 targeted health care students while 17 targeted health professionals. To provide a more detailed characterization of the type and content of the identified intervention studies, we categorized the interventions into eight main types: four targeting students and four targeting health professionals. We categorized the interventions targeting students based on the content of the intervention, whereas we categorized interventions targeting health professionals according to the content and specific health profession (e.g. nurses or medical doctors) because these were often closely related. We present one example of each intervention type, focusing on examples that are illustrative of the intervention types, well-described in the articles, and show a geographical breadth and variation between health care students and professionals. For a detailed description of the intervention studies, see Table 4.

### Interventions targeting students

We identified 47 intervention studies targeting health care students. We categorized these into four different types of interventions: a) Lectures b) Expeditionary interventions c) Contact-based interventions, and d) Observational studies of the effect of attending medical school. Many interventions included a mix of different activities. We divided the interventions according to the most prominent ones. The interventions most often targeted nursing students (*n* = 20) or medical students (n = 20), while six interventions targeted pharmacy students and one targeted a mixed group of students.

#### Lectures

Interventions based on lectures (*n* = 13) were characterized by a teacher-centered approach, and typically took place in a classroom where the teacher provided different educational programs [101, 103, 105, 112, 115–117, 126, 128, 148, 152, 155, 158]. The topics of the lectures varied, including e.g. doctor-patient relationships [101], empathy [103], mental health literacy [152], social distancing [155], fear [148], and knowledge about how patients with mental disorders experience encounters with the somatic health care system [158]. In an example of a classical teaching intervention from Australia, pharmacy students participated in two 12-h Mental Health First Aid courses. The classes addressed themes such as symptoms, evidence-based treatment of several mental disorders, early warning signs of mental disorder, and how to provide initial help to people in a mental health crisis. The courses involved, e.g., case studies and group activities [152].

#### Expeditionary interventions

These interventions (*n* = 17) had in common that they primarily took place outside of the classroom and included clerkships and field trips, e.g. to psychiatric wards [102, 106, 107, 118–120, 129, 131, 139, 141, 147, 149, 153, 156, 157, 159]. The interventions lasted from four hours a week for three weeks [106] to full time for eight weeks [139], and some also included lectures on mental health and psychiatry [106, 118, 119, 139, 157]. While all 17 interventions aimed to reduce stigma, some also investigated the impact on a) students’ interest in psychiatry, b) psychiatry as a career choice, and c) attitudes toward psychiatry.

In some interventions, students visited psychiatric facilities [131] or pharmacies [115], since a visit at local pharmacies allowed pharmacy students to meet patients with mental disorders. Other interventions were mental health camps consisting of a 2–5-day immersive learning program outside of the ‘typical’ clinical setting, where students could meet and interact with people with a mental disorder at camp sites [147, 159]. One of these interventions included students in the United States, who participated in a mental health camp after receiving didactic teaching. The camp consisted of two days working with a group of patients from the local mental health service. The program included trust and confidence-building exercises and socialization through joint preparation of meals and leisure activities. Following the camp, students attended a 15-week mental health placement at either a community facility or a hospital [159].

#### Contact-based interventions

Contact-based interventions (*n* = 12) had in common that they focused on facilitated encounters with patients with mental disorders [110, 123, 125, 127, 130, 132, 133, 142, 146, 151, 154, 164]. These types of interventions were mainly characterized by patients with mental disorders being involved in the lectures, either as educators [130, 151] or as visitors giving testimonies [133, 142, 154]. In some cases, the testimonies were introduced to students via video display [110, 123, 132, 164]. In contrast to interventions based on expeditionary learning, contact-based interventions typically took place in classrooms or other educational settings.

To exemplify, in Spain nursing students participated in a 90-min intervention including testimonies from a mental health professional, a person with a mental disorder and a family member of another person with a mental disorder. They described their experiences with mental disorder, e.g., how the disorder emerged, symptoms and side effects of medication, problems related to family coexistence, and problems in the workplace. Following this, a 30-min discussion among students and the presenters was held [142].

#### Observational studies of the effect of attending medical school

We identified five studies investigating the effect of attending medical school on stigmatizing attitudes and behaviors toward patients with mental disorders [109, 121, 122, 160, 163]. These studies were observational or based on natural experiments in contrast to the other studies. For instance, in Turkey researchers followed freshman medical students from 2008 to 2013. A questionnaire was administered to the participants on their first study year, before receiving any theoretical or practical training on psychiatry. Participants who completed their psychiatry internship were reassessed with a questionnaire five years later [121].

### Interventions targeting health professionals

We identified 17 intervention studies targeting health professionals, including general practitioners and other medical doctors (*n* = 9), emergency personnel (*n* = 3), nurses (*n* = 1), pharmacists (*n* = 2), and non-specific groups of health professionals (n = 2). We categorized these interventions into four intervention types: (1) Interventions targeting general practitioners and medical doctors, (2) Interventions targeting pharmacists, (3) Interventions targeting emergency personnel, and (4) Interventions targeting non-specific groups of health professionals.

#### Interventions targeting general practitioners and medical doctors

Nine studies focused on interventions targeting general practitioners and medical doctors [104, 111, 124, 135, 136, 138, 140, 150, 162]. They focused on attitudes and behaviors toward patients with specific mental disorders such as depression [111, 140, 162] or eating disorders [138]. The interventions differed considerably in content and scope. For example, in Hong Kong general practitioners participated in a 1-year part-time course. The course included 20 interactive seminars on mental disorders and 20 sessions visiting general practitioner consultations, including a written assignment. The seminars were developed and conducted by a family physician and a psychiatrist. After completing the seminars, the participants began clinical attachment in groups [135].

#### Interventions targeting pharmacists

We found two interventions targeting pharmacists (*n* = 2), both of which addressed attitudes and behaviors toward patients with depression [113, 137]. The interventions aimed to empower pharmacists when encountering patients with depression through courses in communication skills, awareness of depression, and use of anti-depressants. For example, Australian pharmacists were taught, by a psychiatrist, a psychologist, and a general practitioner, to give advice and support when dispensing medication. To upgrade their knowledge, the pharmacists received pamphlets on depression [113].

#### Interventions targeting emergency personnel

Few studies (*n* = 3) investigated the impact of educational programs on emergency personnel [143, 144, 161]. These interventions primarily focused on the reception of patients with mental disorders at emergency rooms through courses in evidence-based treatment and communication. For example, in Australia, researchers tested a 2-h lecture focusing on participants’ attitudes and current practice in relation to self-harm. Lectures included theories for understanding self-harm and evidence-based treatment. Teaching material consisted of PowerPoint presentations and short video narratives from clinical practice and consumer reports [143].

#### Interventions targeting non-specific groups of health professionals

Three interventions did not target a specific group of health professionals, but included different professions [108, 134, 145]. These interventions differed considerably in content given that one was a lecture [108], one a workshop [134], and one a stage play [145]. In the latter, researchers from Canada worked closely with an actress and playwright who had bipolar disorder. They developed a one-woman stage play specifically targeting stigma toward that specific disorder. A director was hired for the rehearsal period. The audiences comprised people with bipolar disorder and health care providers working with this target group [145].

## Discussion

The aim of this scoping review was to provide a comprehensive overview of the vast amount of literature within the research field of stigma toward people with a mental disorder in the somatic health care system. In total, we identified 137 studies, which include 73 observational studies and 64 intervention studies. We included qualitative and quantitative studies and a wide range of health care professionals and students. In the analysis, we described the characteristics of the studies and categorized them according to health profession and diagnosis. This contrasts with previous literature reviews that have typically focused more narrowly on, for instance, specific diagnoses or health care professionals ([23, 24, 165–167], e.g.), and only a few previous reviews have had health care students as a target population [168, 169].

This scoping review created a comprehensive overview of the existing literature, employing a broad focus on both health care students and health professionals as well as inclusion of several mental disorders. This broad approach is helpful for identifying important knowledge gaps. All observational studies examined stigmatizing behaviors, attitudes, and/or perceptions among health professionals in the somatic health care system toward patients with mental disorders. We found that most observational studies (*n* = 13) focused on emergency personnel, followed by medical doctors (*n* = 10) and health professionals (*n* = 8), and that most studies (*n* = 40) focused on several diagnoses. In studies focusing on single, specific diagnoses, the most frequent mental disorders were self-harm (n = 10) and schizophrenia (*n* = 9). Finally, we found that most of the observational studies used quantitative methods (*n* = 58), while 13 studies used qualitive methods and only two studies used mixed methods. Considering the complexity of the phenomena, more qualitative- and mixed method studies could deepen our understanding of stigma in somatic health care further.

With this review, we have provided insight into the distribution of studies in relation to the specific health profession and diagnosis that have dominated the literature, and which have been overlooked. We found that only a small number of all the included studies explicitly address stigma toward people with anxiety, bipolar disorder, and borderline personality disorder. This points to a need for future research to explore further the extent and characteristics of somatic health care professionals’ stigmatizing attitudes and behaviors toward patients diagnosed with these disorders.

In general, the content of the included studies spans many different combinations of health professionals and diagnoses, confer Tables 1 and 2. Because of this diversity, it is not possible to conclude whether stigmatizing attitudes and behaviors may be more prevalent among some health care professions compared to others, and whether patients with a specific mental disorder are more exposed to stigmatization than others. However, this could be an interesting theme to investigate further in future research, as this knowledge can develop and strengthen anti-stigma campaigns targeting specific professions within the health sector.

We found that interventions to prevent or reduce stigma toward patients with mental disorders focused either on health care students or health care professionals. We categorized student interventions according to their content and the interventions targeting health professional according to the target group. In total, we identified four types of intervention studies targeting students (including lectures, expeditionary interventions, contact-based interventions, and observational studies of the effect of attending medical school) and four targeting health professionals (including general practitioners/medical doctors, pharmacists, emergency personnel and non-specific health professionals). The interventions varied in content and design, both within and across different target groups. Student interventions generally did not examine a specific mental disorder but rather multiple mental disorders or mental disorders in general, as opposed to interventions for health professionals, which often focused on both a specific target group and a specific diagnosis. This extended focus on intervention studies is, to our knowledge, not seen in previous reviews. Several previous reviews, however, call for more educational interventions to reduce negative attitudes and stigma among health professionals [167, 170], since more education and competency development can be associated with more positive attitudes [23, 171].

Following the scoping review methodology, we did not assess the quality of the studies included in the review, given that we focused on the overview of the literature. Thus, we cannot draw inferences about the effectiveness of interventions toward somatic health care professionals to reduce or prevent stigma. However, we did find that the quality of the effectiveness evaluations varied and that the majority used a quasi-experimental design (with or without a control group), while only eight studies employed an RCT. Therefore, this review points to a need for intervention studies with stronger evaluation designs.

Overall, our scoping review underlines the presence of stigmatizing attitudes and behaviors toward people with a mental disorder in the somatic health care system. The included studies show that stigma may be caused by several factors, e.g. lack of knowledge about mental disorders among health professionals, lack of time to care for more demanding or difficult patients and by health professionals’ experiences of feeling insecure and unsafe in the presence of patients with mental disorders [111, 132, 152]. Following Link and Phelan’s conceptualization of stigma, health professionals’ experiences of lacking knowledge and competencies regarding mental disorders may initiate a stigmatization process in which they—due to dominant cultural beliefs—link undesirable characteristics and negative stereotypes to patients with mental disorders and engage in a separation of “us” from “them”, leading the patients to experience status loss, discrimination and unequal outcomes and opportunities [12]. However, the identified intervention studies reveal that attitudes toward people with mental disorders among somatic health care professionals to a great extent reflect the attitudes of the general population [16, 108, 133]. This way, somatic health care professionals seem to exercise neither more nor less stigmatizing behavior than people in the general population. Importantly, and in contrast to the general population, health professionals possess power to determine the course, type and circumstances of treatment offered to patients with mental disorders. According to Link and Phelan (2001), power is precisely key to understanding stigma, because stigmatization is entirely contingent on access to social, economic and/or political power that allows the different elements of stigma to unfold [12]. Therefore, stigmatizing attitudes and behaviors toward mental disorders among health professionals constitute a critical problem that needs to be addressed both during their education and continuously throughout their professional career as part of their continuing professional development.

### Strengths and weaknesses

This review has several limitations. First, as the search was limited to studies published after 2008, as well as studies in English or Nordic languages, we may have overlooked relevant studies and anti-stigmatizing interventions. Second, as mentioned above and as applies to scoping reviews in general, we did not assess the quality of the included studies.

Despite these limitations, we note several strengths of this study. First, a methodological strength is the fundamental systematic approach to charting the studies. We have systematically selected keywords and searched in selected databases based on several inclusion and exclusion criteria. Additionally, the screening process was undertaken on different levels: by title screening, abstract screening, and full-text screening in the review manager Covidence, after which all included studies were systematically organized and analyzed in NVivo. The use of NVivo contributed to a systematic organization of the included studies, which provided a useful tool for creating overview and high-level systematization of all included studies. Additionally, we consider it a strength that we have included intervention studies from the entire world. Although organization and practice within the somatic health care system are diverse worldwide, which reduces the transmissibility of the interventions, this knowledge contributes to the research field and can further inspire and mobilize new interventions. Finally, we consider it a strength that we have included a consultation element (e.g. stage 6) in our review process, as recommended by Arksey and O’Malley [28].

## Conclusion

This scoping review has contributed to our knowledge about stigmatization of people with mental disorders in the somatic health care system. The large number of studies identified in this review suggests that stigmatizing attitudes and behaviors toward patients with mental disorders, within a somatic health care setting, is a worldwide challenge. By including studies focusing on different health professions and mental disorders, instead of focusing solely on a single profession or diagnosis as seen in many reviews, this review contributes with a more comprehensive overview.

The findings point to a need for further research on stigma toward patients with anxiety, bipolar disorder and borderline personality disorder. Furthermore, it would be beneficial to further knowledge on whether stigmatizing attitudes or behaviors toward mental disorders are more prevalent in some health care professions than others. Such knowledge could contribute to more targeted interventions.

## Supplementary Information

**Additional file 1.**

## Data Availability

All data generated or analyzed during this study are included in this published article. The search strategy is available as an additional file.
