# Supplementary material for: Somatic health care professionals’ stigmatization of patients with mental disorder: a scoping review
Source: BMC Psychiatry. 2021 Sep 7;21:443. doi: 10.1186/s12888-021-03415-8 (PMC8424966; doi:10.1186/s12888-021-03415-8)
Supplement: Supplementary file 1 — Additional file 1. [file 12888_2021_3415_MOESM1_ESM.docx]

# Appendix A

**Medline**

Database version: Ovid MEDLINE(R) and Epub Ahead of Print, In-Process & Other Non-Indexed Citations and Daily 1946 to May 23, 2019

|  | \| **Search terms** \| \| --- \| | \| **Results** \| \| --- \| |
| --- | --- | --- | --- | --- |
| #1 | Mental disorders/ |  |
| #2 | Anxiety Disorders/ |  |
| #3 | "Bipolar and Related Disorders"/ |  |
| #4 | Bipolar Disorders/ |  |
| #5 | Mood Disorders/ |  |
| #6 | Depressive Disorder/ |  |
| #7 | Depressive Disorder, Major/ |  |
| #8 | Personality Disorders/ |  |
| #9 | Borderline Personality Disorder/ |  |
| #10 | Schizophrenia/ |  |
| #11 | Schizophrenia, Paranoid/ |  |
| #12 | Depression/ |  |
| #13 | Self-Injurious Behavior/ |  |
| #14 | Self Mutilation/ |  |
| #15 | Anorexia Nervosa/ |  |
| #16 | Binge-Eating Disorder/ |  |
| #17 | Bulimia/ |  |
| #18 | Anorexia/ |  |
| #19 | Bulimia Nervosa/ |  |
| #20 | "Feeding and Eating Disorders"/ |  |
| #21 | Phobia, Social/ |  |
| #22 | 1 or 2 or 3 or 4 or 5 or 6 or 7 or 8 or 9 or 10 or 11 or 12 or 13 or 14 or 15 or 16 or 17 or 18 or 19 or 20 or 21 | **528254** |
| #23 | (((Mental or behavio?r* or psychi* or personality or mood or depressive or anxiety or schizophrenic or affective or bipolar or eating or manic) adj3 (ill-ness* or disorder* or disease* or diagnos* or disturbance*)) or (Anxiety adj3 (Neuros?s or state)) or psychotic reaction* or (Borderline adj3 (state or per-sonality or disorder*)) or Depression* or psychotic or Schizophrenia* or (Schizophrenic adj3 (syndrome* or reaction*)) or (Depressive adj3 (episode* or state or neuros?s or syndrome)) or Self harm or Self Injur* Behavio?r* or Self injur* or Self Mutilati* or automutilati* or Head Banging or Manic epi-sode* or Self inflicted wound* or self inflicted injur* or Self Destructive Beha-vio?r* or Appetite disorder* or Anorexia or Bulimia or EDNOS or Binge ea-ting or social phobia).mp. |  |
| #24 | 22 or 23 | **904424** |
| #25 | "Attitude of Health Personnel"/ |  |
| #26 | Professional-Patient Relations/ |  |
| #27 | Nurse-Patient Relations/ |  |
| #28 | Physician-Patient Relations/ |  |
| #29 | Dentist-Patient Relations/ |  |
| #30 | 25 or 26 or 27 or 28 or 29 | **236172** |
| #31 | Stereotyping/ |  |
| #32 | Social Perception/ |  |
| #33 | Social Discrimination/ |  |
| #34 | Social Stigma |  |
| #35 | Prejudice/ |  |
| #36 | Social Distance/ |  |
| #37 | Social Marginalization/ |  |
| #38 | Empathy/ |  |
| #39 | 31 or 32 or 33 or 34 or 35 or 36 or 37 or 38 | **76695** |
| #40 | Health Personnel/ |  |
| #41 | Nurses/ |  |
| #42 | Nurse Specialists/ |  |
| #43 | Nurse Clinicians/ |  |
| #44 | Nurse Practitioners/ |  |
| #45 | Family Nurse Practitioners/ |  |
| #46 | Nursing Staff/ |  |
| #47 | Nursing Staff, Hospital/ |  |
| #48 | Nurses, Public Health/ |  |
| #49 | Occupational Therapists/ |  |
| #50 | Physical Therapists/ |  |
| #51 | Physicians/ |  |
| #52 | General Practitioners/ |  |
| #53 | Surgeons/ |  |
| #54 | Physicians, Family/ |  |
| #55 | Physicians, Primary Care/ |  |
| #56 | Dentists/ |  |
| #57 | Medical Staff/ |  |
| #58 | Medical Staff, Hospital/ |  |
| #59 | Emergency Medical Technicians/ |  |
| #60 | Personnel, Hospital/ |  |
| #61 | 40 or 41 or 42 or 43 or 44 or 45 or 46 or 47 or 48 or 49 or 50 or 51 or 52 or 53 or 54 or 55 or 56 or 57 or 58 or 59 or 60 | **324433** |
| #62 | 39 and 61 | **7250** |
| #63 | 62 or 30 | **239903** |
| #64 | (((Stigma* or exclu* or distanc* or discrimin* or marginali* or attitude* or ste-reotyp* or prejud* or Therapeutic process* or Dangerousness or perception* or labeling or compassion* or Sympathy or Empathy) adj6 (((healthcare or health care or nursing or medical) adj2 (provider* or profession* or worker* or practitioner* or staff)) or health practitioner* or family practitioner* or doc-tor* or nurse* or physician* or general practitioner* or physical therapist* or physiotherapist* or Occupational therapist* or surgeon* or clinician* or hospi-tal personnel or rescue personnel or dentist* or (non psychiatr* adj2 consul-tant*) or (Emergency adj2 department adj2 staff) or paramedic* or (Emer-gency adj2 Medical adj2 Technician*))) or ((((healthcare or health care or nursing or medical) adj2 (provider* or profession* or worker* practitioner* or staff)) or health practitioner* or doctor* or nurse* or physician* or general practitioner* or family practitioner* or physical therapist* or physiotherapist*  or Occupational therapist* or surgeon* or clinician* or hospital personnel or rescue personnel or dentist* or (non psychiatr* adj2 consultant*) or (Emer-gency adj2 department adj2 staff) or paramedic* or (Emergency adj2 Medi-cal adj2 Technicians)) adj2 Patient* adj2 Relation*) or Diagnostic overshado-wing).mp | **152341** |
| #65 | 63 or 64 | **265743** |
| #66 | 24 and 65 | **21424** |
| #67 | limit 66 to (yr="2008 -Current" and (danish or english or norwegian or swedish)) | **7304** |
|  |  |  |
|  |  |  |
|  |  |  |
|  |  |  |
|  |  |  |
|  |  |  |
|  |  |  |
|  |  |  |
|  |  |  |
|  |  |  |
|  |  |  |
|  |  |  |
|  |  |  |
|  |  |  |
|  |  |  |
|  |  |  |
|  |  |  |
|  |  |  |
|  |  |  |
|  |  |  |
|  |  |  |
|  |  |  |
|  |  |  |
